# Human-computer interactions with farm animals—enhancing welfare through precision livestock farming and artificial intelligence

**DOI:** 10.3389/fvets.2024.1490851

**Published:** 2024-11-14

**Authors:** Suresh Neethirajan, Stacey Scott, Clara Mancini, Xavier Boivin, Elizabeth Strand

**Affiliations:** ^1^Faculty of Agriculture and Computer Science, Dalhousie University, Halifax, NS, Canada; ^2^School of Computer Science, University of Guelph, Guelph, ON, Canada; ^3^The Open University Milton Keynes, Nottingham, United Kingdom; ^4^Université Clermont Auvergne, INRAE, Saint-Genès Champanelle, France; ^5^College of Social Work and College of Veterinary Medicine, University of Tennessee, Knoxville, TN, United States

**Keywords:** precision livestock farming, animal-computer interaction, artificial intelligence, farm animal welfare, human-computer interaction, one welfare, sensor technology, sustainable agriculture

## Abstract

While user-centered design approaches stemming from the human-computer interaction (HCI) field have notably improved the welfare of companion, service, and zoo animals, their application in farm animal settings remains limited. This shortfall has catalyzed the emergence of animal-computer interaction (ACI), a discipline extending technology’s reach to a multispecies user base involving both animals and humans. Despite significant strides in other sectors, the adaptation of HCI and ACI (collectively HACI) to farm animal welfare—particularly for dairy cows, swine, and poultry—lags behind. Our paper explores the potential of HACI within precision livestock farming (PLF) and artificial intelligence (AI) to enhance individual animal welfare and address the unique challenges within these settings. It underscores the necessity of transitioning from productivity-focused to animal-centered farming methods, advocating for a paradigm shift that emphasizes welfare as integral to sustainable farming practices. Emphasizing the ‘One Welfare’ approach, this discussion highlights how integrating animal-centered technologies not only benefits farm animal health, productivity, and overall well-being but also aligns with broader societal, environmental, and economic benefits, considering the pressures farmers face. This perspective is based on insights from a one-day workshop held on June 24, 2024, which focused on advancing HACI technologies for farm animal welfare.

## Introduction

The concept of animal welfare has significantly evolved over recent decades, with a growing emphasis on the importance of considering the well-being of individual animals, rather than solely focusing on groups (1). This shift is particularly pertinent in the domains of cattle and swine farming, where the advent of advanced technologies has made individualized care increasingly feasible. However, in the realm of poultry farming, welfare management still predominantly relies on decisions made at the flock level, which presents significant challenges for ensuring the well-being of each bird (2). Animal-Computer Interaction (ACI) is an emerging field that explores the interaction between animals and technology, aiming to enhance animal welfare and understanding through innovative interfaces. Here we focus on the integration of Human-Computer Interaction (HCI) (3) and ACI (4) in the context of farm animal welfare. We examine the current economic emphasis on productivity while advocating for a shift toward animal-centered farming practices. ACI, a rapidly growing field, enhances HCI by including animals as active technology users, aiming to tailor technologies to their specific needs and behaviors. This approach requires a robust methodological and ethical framework that acknowledges animals as vital stakeholders in technology design. Insights from successful companion animal technologies provide valuable lessons for adapting these innovations to improve farm animal welfare.

## Materials and methods

A one-day workshop on Human-Computer Farm Animal Interactions (HCFAI) was held on June 3, 2024, co-organized by Dalhousie University and the University of Guelph (5). Led by Professor Suresh Neethirajan, the workshop aimed to explore the integration of digital innovation with animal care in modern farming practices. The event brought together over 150 participants from 18 countries, representing a diverse range of stakeholders, including researchers, technology developers, farmers, and industry representatives.

The workshop featured keynote presentations by leading experts in animal welfare and smart farming technologies, followed by panel discussions with industry leaders and researchers. Interactive sessions facilitated collaborative problem-solving and idea exchange. The main topics addressed during the workshop included current applications of HCFAI technologies in farm settings, ethical considerations related to implementing digital technologies for animal welfare, and the challenges and opportunities of adapting companion animal technologies for farm use. The discussions also focused on future research and development directions in HCFAI. All proceedings were documented through audio and video recordings, and designated rapporteurs took detailed notes. Key points and conclusions from these discussions were compiled and summarized for further analysis, providing valuable insights into the current state and future potential of HCFAI technologies.

## Literature review methodology

Following the workshop, a comprehensive literature review was conducted to contextualize and expand upon the insights gained during the event. The literature search was carried out using the databases Web of Science, Scopus, and Google Scholar. The search included combinations of keywords such as “precision livestock farming,” “animal-computer interaction,” “farm animal welfare,” and “digital agriculture.” Inclusion criteria for the review were: peer-reviewed articles published between 2018 and 2024, studies focusing on the application of digital technologies in farm animal welfare, and research addressing ethical considerations in animal-computer interactions. The literature review process was conducted independently of the workshop participants, and participants did not have the opportunity to review the manuscript prior to its completion.

### Current state of HACI technologies in farm animal welfare

The current landscape of HACI technologies spans a spectrum from widely implemented systems to experimental technologies and future concepts. In commercial settings, automated milking systems have become commonplace in dairy farms, allowing cows to be milked on demand, which has been shown to reduce stress and improve overall welfare (6–8). Similarly, wearable health monitors, such as RFID tags and accelerometers, are now routinely used in cattle and swine farms to track individual animal health and behavior (9–13). These technologies represent the current state-of-the-art in commercially available HACI solutions.

Moving beyond established technologies, several promising innovations are currently in experimental stages. Facial recognition for emotion assessment in dairy cows and pigs has shown potential in laboratory settings but has not yet been widely implemented on commercial farms (14–17). Similarly, vocalization analysis systems for interpreting pigs, chicken and cattle vocalizations are being developed, though these remain primarily in the research phase (18–21). These experimental technologies represent the cutting edge of HACI research and development, with the potential to significantly enhance our understanding of animal welfare in the near future.

Looking ahead, the future of HACI technologies holds exciting possibilities. In poultry farming, where current technologies primarily focus on flock-level management, future systems may enable individual bird monitoring and care, representing a significant leap forward in welfare management for this sector. Additionally, advanced interactive enrichment devices, inspired by technologies developed for companion animals, are being conceptualized to provide cognitive stimulation for farm animals (22–24). While still in the early stages of development, these future technologies have the potential to revolutionize farm animal welfare by providing unprecedented levels of individualized care and enrichment.

### Precision livestock farming and AI in cattle and swine

Precision Livestock Farming (PLF) employs advanced technologies such as Artificial Intelligence (AI), sensors, and big data analytics to monitor and manage the health indicators, behavioral patterns, and welfare of farm animals as well as production processes (25, 26). In dairy farming, innovations like automated milking systems, wearable health monitors, and real-time data analysis have revolutionized the care of individual cows. Commonly used technologies include RFID tags and accelerometers, which provide data on cow identification, location, and activity levels (27, 28). Accelerometers detect movement patterns, enabling early identification of issues like lameness or abnormal behaviors that may indicate stress or illness (29, 30). Rumination monitors assess chewing activity to evaluate digestive health and detect potential metabolic disorders (31).

While routine measurements of physiological signals such as heart rate, respiration rate, and thermal signatures are not yet common practice and have been explored primarily in academic studies, there is significant potential for incorporating real-time monitoring of these parameters as sensor technologies advance (32). AI plays a crucial role in analyzing this wealth of data. Predictive health analytics powered by AI algorithms can identify subtle changes that precede clinical signs of disease. For example, machine learning models process complex datasets to predict the onset of mastitis in dairy cows by recognizing patterns in milk yield fluctuations, conductivity, and somatic cell counts (33). In swine farming, AI systems analyze vocalizations, feeding behavior, and environmental data to detect respiratory illnesses or heat stress before they escalate (34, 35).

Similarly, swine farming has seen substantial improvements in individual animal care through the use of smart feeding systems, health monitoring devices, and environmental sensors. Automated feeding systems are calibrated to deliver feed tailored to each pig’s weight and health condition, ensuring optimal nutrition (36). AI applications enable precision feeding strategies by adjusting feed composition and quantity based on individual growth rates and nutritional requirements, optimizing feed efficiency and reducing waste (37). While PLF technologies like RFID tags and accelerometers are already employed for monitoring livestock health, indirectly measuring anomalies of behaviors (aggression, social interactions, etc.) and productivity, the integration of Animal-Computer Interaction (ACI) principles to actively enhance welfare is still developing, especially in areas like emotional well-being and enrichment (38). We propose the integration of advanced AI algorithms for real-time behavioral analysis, which can identify subtle changes in animal behavior indicative of stress or illness, thus addressing current gaps in early detection and welfare management. By incorporating more sophisticated AI-driven analytics, farmers can implement proactive health management practices, enhance animal welfare, measure affective states, and improve overall farm productivity (39, 40).

### Challenges in poultry welfare management

Despite technological advances, poultry farming continues to face unique welfare challenges. The vast number of birds and the reliance on flock-level decision-making complicate the task of ensuring individual bird welfare. Existing technologies, which often average data across large groups, can obscure individual health and welfare issues. There is a pressing need for further research and development of technologies that can provide individualized care, potentially through the use of computer vision systems (41) or other tracking techniques (42) to monitor each bird’s health and behavior comprehensively.

Furthermore, the specific physical and behavioral needs of poultry, such as requirements for perching, dust bathing, and foraging, differ markedly from those of larger farm animals. Creating environments that cater to these needs on a large scale poses substantial challenges. Technologies capable of detecting behavioral changes, like reduced movement or alterations in vocalization patterns, are crucial for early identification of welfare issues, thus necessitating a nuanced approach to technology implementation in poultry farming (43, 44).

### Economic emphasis on productivity

Farmers often find themselves at the crux of balancing productivity with animal welfare, as economic drivers emphasize maximizing output. While PLF and AI can boost productivity by optimizing feed, health, and environmental conditions, it is crucial that these technologies also prioritize animal welfare (13, 45). A paradigm shift toward animal-centered farming is emerging as a method of both prioritizing animal well-being and economic productivity through targeted farm management approaches and techniques (46, 47). The economic pressures on farmers are substantial. They must balance the need for efficient food production with the costs of adopting new technologies, which may create reluctance to invest in systems that do not offer immediate financial returns. However, evidence suggests that improving animal welfare can yield long-term economic benefits, including enhanced health and productivity, lower veterinary costs (48), and increased public trust in the farm industry’s ability to attend to consumer demand for ethically produced goods (49).

Work efficiency is crucial for overall farm productivity. Ergonomically, farm work health is defined by the discrepancy between workers’ expectations and their actual working conditions; reducing this gap is essential for improving job satisfaction and motivation (50). Managing handling times and worker presence significantly impacts both human and animal stress levels, influencing both animal and human welfare, health, reproduction, and overall production (51, 52). The integration of digital technologies can streamline routine tasks such as monitoring, feeding, and handling, reducing physical fatigue and time pressure on farm workers, thereby enhancing their job satisfaction and productivity (53).

### Integrating companion animal technologies to promote animal welfare

The discipline of ACI has markedly improved companion animal welfare through innovative technologies, offering significant insights for adapting these advancements to farm animal care (54). While these technologies can be beneficial, it is essential to be cautious of their potential adverse effects observed in companion animals to ensure they are suitably adapted for farm settings (55).

Interactive devices, such as toys managed through smartphone applications, have revolutionized how we interact with companion animals (56). Integrating features like cameras and treat-dispensing mechanisms can not only provide physical stimulation but also strengthen emotional bonds between pets and their owners, even when they are not in the same room (57, 58). Translating this technology to farm environments, interactive feeders could encourage natural behaviors such as foraging and rooting, enriching the lives of farm animals like cows and pigs, and seamlessly integrating with essential farm operations.

Similarly, fitness trackers customarily used for pets provide a detailed account of physical activity, sleep patterns, and caloric expenditure. These trackers offer customized exercise recommendations that can enhance pet health and vitality (59, 60). For farm animals, adopting similar technology could facilitate monitoring of individual health and activity levels. As discussed above, currently utilized accelerometers are pivotal in early detection of conditions such as lameness and stress-induced behaviors (61, 62).

Furthermore, automated feeders and water dispensers ensure pets receive consistent and tailored nutrition and hydration in the absence of their owners. Companion animal feeders often track not only the amount of feed consumed but also the timing, frequency, and patterns of feeding. Adapting these advanced monitoring capabilities to farm settings could help detect early signs of health issues, such as decreased appetite or changes in eating patterns, which are not always captured by current farming sensor systems focusing primarily on quantity (63). This level of detailed monitoring could significantly enhance individual farm animal health management.

By connecting feeders to wearable devices and environmental sensors, we can create a comprehensive monitoring system. For instance, adjusting feed formulations in real-time based on an animal’s activity levels, stress indicators, or environmental conditions could optimize health and productivity more effectively than current practices (64). While individual feeding systems are already in use for swine farming (65), similar advancements are less prevalent in dairy cattle and virtually non-existent in poultry farming due to the challenges of individual care. We propose adapting these advanced feeders to other species, addressing their specific nutritional and behavioral needs, and overcoming the limitations of current group-based feeding systems (66).

### Incorporating AI algorithms for enhanced farm animal welfare

Incorporating AI algorithms to analyze feeding data can help predict health issues before they become clinically apparent (67, 68). This proactive approach aligns with precision farming’s goal of individualized care. Growing research attention suggests that cognition of these species is more complex than originally thought (69, 70) and reducing stress as well as providing for these animals’ biological and social needs can have positive effects for both welfare and economics (71). By integrating advanced monitoring, cognitive enrichment, and AI-driven analytics, next-generation automated feeding systems that do more than adjust feed based on basic metrics could significantly enhance animal welfare and farm efficiency.

### Pet chat gadgets and their agricultural applications

Pet chat gadgets represent another area of innovation, allowing pet owners to maintain visual and auditory communication with their pets through video calls facilitated by integrated screens and cameras (72). Adapting such technology for agricultural use could revolutionize how farmers engage with and monitor their livestock, especially in expansive farm settings or remote locations, ensuring timely interventions and continuous welfare monitoring (73). However, while entertainment apps for pets often simulate prey movements to engage their predatory drives (74), adapting such applications for farm animals requires a thoughtful redesign. Farm animals, being primarily prey, exhibit different natural behaviors. For example, interactive applications for these species could instead focus on enhancing cognitive enrichment through activities that simulate puzzle-solving and foraging, which resonate with their natural behaviors (75–77) (Table 1).

**Table 1:** Overview of current ACI devices for companion animals—data collected and behaviors monitored.

ACI device type	Data collected	Behaviors monitored	References
Interactive toys	Activity levels, play patterns	Engagement, exercise habits	(56)
Smart feeders	Feeding times, portion sizes	Eating habits, appetite changes	(23, 60, 165)
Wearable health monitors	Heart rate, temperature, activity levels	Overall health, stress levels, sleep patterns	(166)
GPS trackers	Location, movement patterns	Roaming habits, activity levels	(167)
Video chat devices	Visual and audio data	Social interaction, separation anxiety	(168)
Automated litter boxes	Waste output, usage frequency	Bathroom habits, potential health issues	(169–172)
Emotion recognition cameras	Facial expressions, body language, vocalizations	Emotional states, stress levels	(72, 173–178)
Smart water dispensers	Water consumption, drinking patterns	Hydration levels, potential health issues	(63, 179)
Interactive toys	Activity levels, play patterns	Engagement, exercise habits	(180–182)

### Enhancing farm animal welfare ~ advanced applications of HACI

Integrating HACI into farm animal welfare represents a paradigm shift in traditional agricultural practices, leveraging advanced technologies to address complex welfare challenges. Artificial intelligence (AI)-driven predictive health management, utilizing techniques such as deep learning and pattern recognition, allows for the early identification and mitigation of subclinical health issues, thereby enhancing individual animal health and overall farm efficiency (78). Furthermore, HACI facilitates the development of interactive enrichment devices grounded in ethological principles, simulating natural behaviors such as foraging and social interactions to reduce stress and promote positive affective states (79). For instance, automated foraging systems encourage pigs to engage in rooting behaviors; interactive platforms for dairy cows enhance social bonding and mitigate isolation-induced stress; and automated dust-bathing stations for poultry promote natural bathing behaviors, thereby improving flock well-being (80–82).

### Advanced monitoring in poultry farming

In poultry farming, achieving individualized care is challenging due to the scale of operations. However, HACI technologies, particularly environmental sensors, are transforming welfare management at the flock level. These sensors continuously monitor variables such as temperature, humidity, ammonia concentrations, air quality, and light levels within poultry houses, striving to maintain conditions that optimize flock health and productivity (83). When combined with imaging and proximity sensor data and integrated with feed and water management systems, it becomes possible to assess the individual needs of each bird. AI-driven algorithms analyze this comprehensive dataset in real time, enabling the detection of subtle behavioral changes and stress indicators that standard automated systems might overlook (84, 85). For instance, AI can integrate temperature trends, humidity fluctuations, and ammonia concentrations to anticipate respiratory problems, enabling preemptive ventilation adjustments before conditions become harmful (86). Additionally, AI identifies variations in feeding patterns, movement, and social interactions to understand the cognitive capabilities and emotional states of the birds, allowing for tailored interventions such as adjusting lighting to reduce stress or modifying feed composition to meet specific nutritional requirements.

### Applications of HACI in genetic selection and breeding programs

HACI technologies have the potential to open new frontiers in genetic selection and breeding by enabling the precise measurement of complex phenotypes that were previously difficult to record at scale. These advancements allow for more welfare-oriented breeding strategies, integrating behavior and health data with traditional productivity metrics (87). Tail biting, a behavior with a genetic basis, significantly affects both animal welfare and farm productivity. However, monitoring this behavior in large-scale operations has posed challenges. HACI technologies, particularly computer vision and machine learning algorithms, now offer the ability to track and monitor individual pigs’ behaviors, including tail-biting tendencies (88). For instance, D’Eath et al. (89) developed a 3D video system that detects changes in tail posture, which are early indicators of tail biting. Integrating this data into genetic evaluations allows breeding programs to select against these damaging behaviors, thus reducing the prevalence of tail biting in future pig generations. In loose-housed dairy systems, HACI can provide deeper insights into social dynamics and disease spread. Proximity sensors and network analysis tools can map cow-to-cow interactions, identifying potential “super-spreaders” within a herd. This information can be integrated into breeding programs to select for animals with reduced tendencies for disease transmission. For example, Chopra et al. (90) demonstrated how proximity loggers can analyze cattle contact networks, enabling the identification of individuals with high contact rates. Incorporating such data complements current selection criteria for disease resistance, providing a more comprehensive approach to disease management in breeding (91).

### Promoting positive affective states

Advancements in Human-Animal Computer Interaction (HACI) technologies are significantly improving the ability to measure positive emotional states in farm animals, a key aspect of welfare-oriented breeding. For example, Proctor and Carder (2014) demonstrated that specific ear postures in dairy cows are linked to positive, low-arousal states (92). By utilizing machine learning algorithms to analyze ear movements captured through high-speed cameras, HACI systems can now quantify these emotional experiences on a large scale. This data can then be integrated into breeding programs to select animals with greater propensity for positive welfare states, advancing both animal welfare and productivity over time (93).

The implementation of HACI in breeding programs represents a shift toward more welfare-centric agricultural practices. By selecting for animals that display reduced harmful behaviors, such as tail biting, alongside enhanced disease resilience and a stronger capacity for positive emotional states, we can foster more sustainable and ethical farming systems (94). In addition to genetic selection, HACI technologies, coupled with AI, are instrumental in promoting positive affective states by creating tailored environments that meet the specific behavioral needs of poultry, dairy cows, and pigs. For instance, in poultry, automated perching systems with motion sensors activate roosting structures to encourage natural behaviors, while dust bathing stations adjust humidity and dust levels to optimize comfort and engagement. AI-controlled foraging devices release food pellets at variable intervals, simulating natural foraging behaviors and enhancing cognitive engagement.

### Technological innovations in animal communication

Recent advancements in technologies such as natural language processing and acoustic analysis are significantly enhancing our understanding of farm animals. These tools convert animal vocalizations into actionable insights, enabling a nuanced interpretation of emotional states and related needs (95). For instance, an automated recognition system has been developed to monitor pig welfare on-farm by classifying vocalizations based on emotional valence and context (96, 97). Additionally, machine learning algorithms have been applied to analyze dairy cattle vocalizations, effectively identifying signs of distress and isolation (98). Recent preprints have further demonstrated the potential of these technologies; one study utilized a Natural Language Processing-based model to decode chicken vocalizations, enabling the assessment of their emotional states and facilitating non-invasive welfare monitoring (20). Another investigation employed Convolutional Neural Networks to analyze stress-induced vocalization patterns in laying hens, highlighting the ability to detect stress responses through audio analysis (21). These advancements illustrate how AI-driven technologies enhance the ability to monitor and interpret animal vocalizations in real time, leading to more proactive and informed management practices.

### Utilizing facial recognition to assess affective states

Originally developed for pets (99, 100), facial recognition technology is now being adapted to farm settings, particularly for dairy cows and pigs (14–17, 101). These systems analyze facial expressions to assess emotions, providing a non-invasive way to gage an animal’s mood and well-being. This application is particularly promising as it offers a direct method to monitor the emotional health of farm animals, facilitating improved welfare practices based on reliable, real-time data.

### Efficiency and scalability of HACI systems

The development of animal behavior analysis algorithms has traditionally been hindered by the time-consuming and resource-intensive process of data annotation. However, advancements in artificial intelligence and machine learning have dramatically transformed this landscape, offering scalable solutions that significantly reduce the manual burden (102).

AI-driven tools offer efficient methods to address the challenges associated with data annotation by integrating several advanced techniques. Semi-automated annotation systems, for instance, utilize pre-trained models to suggest labels, which human annotators can verify or adjust, streamlining the process while maintaining high accuracy (103). Additionally, active learning frameworks prioritize the most informative data points, minimizing the overall amount of manual labeling required for effective model training (104). Transfer learning techniques further enhance efficiency by leveraging models trained on extensive datasets, enabling accurate outcomes with fewer annotations in specific applications (105). Moreover, synthetic data generation provides realistic datasets to complement real-world data, thereby reducing the need for labor-intensive manual annotation (106). Automated quality control systems also play a critical role by detecting inconsistencies in annotations, ensuring high data integrity with reduced human intervention (107). Together, these AI-driven innovations significantly alleviate the manual workload and optimize the annotation process.

Advanced platforms like CVAT, DataLoop, and Labelbox integrate AI-powered features, enabling automated identification of basic animal behaviors—such as standing, sitting, or lying—significantly accelerating the annotation process. Moreover, when novel breeds or environments are introduced, transfer learning and domain adaptation allow existing models to adapt with minimal additional data (108). This evolution is also reshaping the role of human annotators. Instead of performing repetitive tasks, they are now engaged in more intellectually stimulating activities, such as quality control, managing complex behaviors, and refining AI-generated annotations (109). This shift not only improves the efficiency of the annotation process but also enhances job satisfaction, addressing concerns about monotonous labor. In the MooAnalytica research group of Dalhousie University, researchers adopt these AI-assisted techniques to optimize the annotation process, reducing both time and costs while maintaining high accuracy. By employing these advanced tools, they efficiently manage large datasets across various breeds and environments, illustrating that annotation is no longer a limiting factor in scaling HACI technologies.

### Broadening the scope of HACI in farm settings

The integration of HACI in farm settings opens up new avenues for enhancing the welfare of farm animals. This innovative approach goes beyond meeting the physical needs of the animals, focusing also on enriching their emotional well-being. HACI technologies facilitate better emotional interactions between humans and animals and improve our understanding of animal behavior. By leveraging these technologies, we can support natural behaviors through interactive devices and environments tailored to encourage species-specific activities, such as foraging and social interaction, which are essential for the mental health of farm animals.

### Moving toward animal-centered farming

Adopting an animal-centered approach requires a paradigm shift in farming that equally prioritizes animal welfare and economic productivity. HACI technologies can facilitate this transition by providing innovative tools that enhance animal well-being and enable precise monitoring of welfare indicators (110, 111). Recent studies have demonstrated the efficacy of advanced environmental enrichment strategies and sensor-based technologies in reducing stress levels and improving overall welfare in farm animals. Oliveira et al. (2020) examined various environmental enrichment strategies for nursery piglets, evaluating different types of enrichment objects and their effectiveness in maintaining pig interest and reducing stress-related behaviors (112). This research highlights the ongoing development of sophisticated enrichment methods beyond simple toys, demonstrating the potential for more advanced HACI applications.

In the realm of dairy cattle, automated milking systems equipped with sensors and data analytics allow cows to choose when to be milked, promoting natural behaviors and reducing stress associated with fixed milking schedules (113). The integration of Internet of Things (IoT) technologies in animal farming has shown promise in enhancing both animal welfare and operational efficiency (114). Sensor technologies can monitor animal behavior and health, providing real-time data to improve welfare and productivity. This application of modern technology in farm animal care goes beyond basic enrichment methods, offering more comprehensive solutions.

Furthermore, a study exploring the transformation of adaptation physiology in farm animals through sensors reviews the potential of the latest technologies to be used as assessment tools for measuring mental, behavioral, and physiological states in real time (9). These advanced technologies provide more comprehensive and precise monitoring of animal well-being compared to traditional methods, enabling detailed tracking of how enrichment tools impact animal behavior and emotional states. By leveraging these HACI technologies, farmers can assess the short- and long-term effects of enrichment strategies on animal activities, emotional states, and behaviors, including social interactions, frustration, and stereotypies. This data-driven approach allows for strategic adjustments to optimize outcomes, providing a more nuanced and effective method for enhancing animal welfare compared to traditional enrichment methods.

### Economic impact and consumer demand

The deployment of HACI technologies in farm animal welfare is not only an ethical imperative but also a strategic economic decision. Improved welfare standards lead to better animal health and increased productivity, which in turn can significantly reduce veterinary costs and enhance farm profitability (48). There is also a growing consumer demand for products from farms that prioritize animal welfare (115, 116), reflecting a broader trend toward ethical consumption habits. Farms that adopt HACI technologies are well-positioned to meet this demand, thereby gaining a competitive edge in the market.

Furthermore, the benefits of improved animal welfare extend beyond immediate financial gains. Healthier animals typically exhibit better growth rates, enhanced reproductive performance, and increased overall efficiency (117). These advantages contribute to long-term economic sustainability, aligning farm operations with consumer expectations for food produced in ways that are ethical and environmentally sustainable (118). Government incentives and industry support are vital in this transition, providing the necessary backing to encourage farmers to adopt innovative welfare-enhancing technologies (119).

### Economic considerations and goal conflicts in HACI implementation

The implementation of HACI technologies in farm animal management introduces the need to pursue complex balance between economic feasibility and the enhancement of animal welfare. While these systems offer significant potential to improve welfare, they also require substantial financial investment, leading to potential conflicts between short-term economic constraints and long-term welfare benefits.

In the short term, the initial costs of HACI technologies are substantial, encompassing the purchase of hardware and software, staff training, operational adjustments, and maintenance. These expenses can be prohibitive for farms operating with tight margins, particularly small or mid-sized operations. However, over the long term, HACI technologies have the potential to offset these costs by improving operational efficiency, reducing labor, and lowering disease incidence, resulting in substantial economic returns over time.

An important consideration is the opportunity cost—funds used for HACI could alternatively support traditional welfare measures such as increased staffing or expanded living spaces. Edwardes et al. (2024) emphasize the importance of quantifying the trade-offs between precision livestock farming technologies and conventional welfare improvements, particularly in areas such as mobility management for dairy cattle (120).

The break-even point for HACI technologies varies by farm size and operational type. For instance, Kimmerer et al. (121) found that robotic milking systems reached their break-even point at herd sizes of 60 cows or more, while farmers of smaller herds faced longer periods before realizing economic returns. For more experimental HACI technologies, this break-even point is less predictable and may take years to materialize.

Several studies have demonstrated the economic value of welfare improvements. For example, Jones et al. (2020) found that certain types of environmental enrichment for broiler chickens improved feed conversion ratios and reduced mortality, potentially offsetting implementation costs (122). Similarly, Mozaffari and Nouman (123) showed that welfare-enhancing technologies in the dairy industry can lead to higher milk production and greater farm efficiency.

The decision to adopt HACI technologies must carefully balance economic factors with welfare goals. While the economic justification for these technologies may not apply uniformly across all farm types, evidence suggests that, when properly implemented, HACI can deliver both welfare enhancements and long-term economic benefits. As consumer demand for higher welfare standards increases, investments in HACI may become essential for farms seeking to maintain market competitiveness.

### Collaborative research and development in HACI for farm animal welfare

The effective integration of HACI into farm animal welfare necessitates a collaborative approach that harnesses the unique strengths of researchers, technology developers, multispecies interaction designers, and farmers. Researchers provide critical insights into animal behavior and needs, essential for developing relevant technologies. Technology developers utilize these insights to create practical tools, while farmers implement and provide feedback based on real-world applications. Collaboration with government and industry is crucial for mitigating financial risks, making investments in advanced technologies more appealing. HACI integration also has the potential to increase consumer trust by providing new forms of biological and animal behavioral data, which may be compelling for consumer education about animal handling and welfare practices.

Working across disciplines, professions, and producer-consumer lines presents significant challenges. Advancing HACI in farm animal welfare requires a collective commitment to the knowledge, skills, and attitudes (KSAs) of collaboration. The Science of Team Science (SciTS) field offers models for cross-disciplinary collaboration. One such model, identified by Brasier et al. (124), outlines five competency domains crucial for high-performance translational teams: affect, communication, management, collaborative problem-solving, and leadership. Within these domains, 15 specific KSAs are identified, including psychological safety, knowledge sharing, and situational leadership. This model emphasizes team-emergent skills that develop through practice and interaction, providing a framework for understanding and enhancing team performance in translational science contexts.

Integrating public perspectives through participatory approaches can facilitate dialog between experts and consumers, influencing public perception and policy (125). Developing cost-effective technologies demonstrating tangible benefits to producers, animals, and consumer trust is more likely to encourage the agricultural sector to embrace HACI innovations. This commitment to a collaborative framework is essential for advancing humane and scientifically informed farming practices. This approach promises to transform farm environments, ensuring that animal welfare and productivity are integrated components of modern agricultural practices.

### Discussion—expanding the scope of HACI in farm animal welfare

The integration of HACI technologies into farm animal welfare is not just a progressive step but a transformative movement reshaping how welfare is approached and implemented. By focusing on the individual welfare of animals and addressing the unique challenges in sectors like poultry farming, HACI can establish new standards for enhancing both animal well-being and operational productivity and efficiency.

Collaboration among HACI researchers, multispecies interaction designers, technology developers, and farmers is indispensable for creating tailored solutions that meet the distinct needs of farm animals. This synergy is essential for developing technologies that improve health, reduce stress, and significantly enhance the quality of life for farm animals. Particularly in dairy and swine farming, where individualized care is more feasible, the deployment of technologies like wearable health monitors and automated feeding systems not only streamlines care but also ensures that animals are maintained in optimal condition.

The shift toward animal-centered farming requires a change in perspective from farmers, balancing economic productivity with animal welfare. This paradigm shift, facilitated by HACI technologies, can help develop farming systems that are not only sustainable but also more humane. This approach aligns with growing consumer demands for ethically produced food and enhances the marketability and reputation of farm products (126, 127).

Incorporating advanced technologies into farming practices must adhere to ethical standards that prioritize animal welfare. This involves designing technologies tailored to the specific needs and behaviors of animals, ensuring that these innovations support welfare without causing distress (128). The principle of animal-centered design is crucial—it emphasizes creating technologies that are accessible and effective for animals, enhancing their interaction with these systems (129). It is crucial to extend ethical considerations to how technologies are deployed within farming systems. This includes assessing the long-term impacts of these technologies on animal welfare and ensuring that the data collected from animals are used responsibly (130, 131). Moreover, addressing potential power imbalances between humans and animals is essential, ensuring that all practices promote the well-being of both (132, 133).

The concept of ‘One Welfare’ highlights the interconnectedness of animal welfare with human welfare and broader environmental and socio-economic contexts (134, 135). This perspective is vital when introducing new technologies in farming, as it necessitates the development of new competencies while preserving essential stockmanship skills. Recognizing the diversity in farmers’ attitudes toward animals—from viewing them as partners to seeing them as units of production—is critical for successfully integrating new technologies (136–138). These technologies can either enhance engagement with the profession or, if not carefully implemented, lead to disengagement and harm human-animal relationships. Studies suggest that relational practices could facilitate the use of new technologies throughout the animal’s life cycle (139, 140).

Similar concerns have been expressed by researchers examining technologies designed for companion animals. For example, remote human-dog interaction applications that allow humans to feed or talk to their dogs while they are alone at home could encourage owners to leave their dogs at home for even longer periods, exacerbating the very issue the technologies were designed to alleviate (141, 142). Another example involves health monitoring devices for companion cats, which may lead owners to misinterpret their animals’ health conditions and even disregard veterinary advice (143, 144).

Automating all aspects of everyday interactions with animals may lead to deskilling livestock professionals’ expertise in animal care. Further, elimination of direct or regular contact with animals may lead to “dehumanization” of animals and degradation of moral concern for them (145, 146). These pitfalls are likely to affect technologies designed for farm animals as well. Therefore, it is imperative that such technologies are designed to complement, rather than substitute, human-animal interactions, and to support human-animal relations. This requires input from the various human caregiving roles that are essential to ensuring animals’ welfare is truly prioritized (147).

### Ethical considerations in HACI implementation

The integration of HACI technologies in farming raises important ethical questions that must be critically evaluated through multiple ethical frameworks: consequentialism, deontology, and virtue ethics.

From a consequentialist perspective, the ethical justification for HACI hinges on its outcomes. If AI-driven systems reduce animal suffering, improve welfare, and enhance farm productivity, they can be considered ethically positive. However, this view requires a careful assessment of potential unintended consequences, such as diminishing human-animal interaction or fostering over-reliance on technology, which could undermine the moral and emotional responsibilities farmers have toward their animals (148). In contrast, deontological ethics emphasizes the moral duties and rights involved, independent of outcomes. HACI technologies may challenge the ethical boundaries of how animals are instrumentalized, especially through constant surveillance. If these technologies are used to treat animals as mere tools for production, they may violate the ethical principle of respecting animal autonomy. However, when framed as tools to uphold our duty of care, ensuring that animals receive the best possible conditions, HACI can be ethically permissible.

From a virtue ethics standpoint, the use of HACI can either cultivate or erode virtues like compassion, responsibility, and respect for nature. The moral character of those who design and implement these technologies plays a critical role in determining whether HACI enhances humane treatment or detaches us from the ethical responsibility of being good stewards of animal welfare (149, 150). The intersection of animal rights and welfare brings additional complexity. While some ethicists argue that any use of animals for human benefit is inherently unethical (151, 152), a more moderate animal welfare perspective holds that if HACI technologies demonstrably improve the quality of life for farm animals without perpetuating harmful practices, their ethical implementation is justified (153).

Furthermore, environmental ethics cannot be overlooked. While HACI technologies may offer individual animal welfare benefits, their environmental footprint—through energy consumption and electronic waste—may counteract these gains. Ethical implementation requires careful consideration of both animal welfare and environmental sustainability (154). Another important dimension is transparency and informed consent. While animals cannot provide informed consent, our ethical obligation extends to ensuring that these technologies are used in ways that do not violate their welfare. Moreover, consumers have a right to know how these technologies are applied in food production, raising questions about how to effectively communicate HACI use in farming practices (155, 156). Finally, balancing stakeholder interests—animals, farmers, consumers, and the ecosystem—presents an ethical challenge. HACI must be implemented in a way that promotes animal welfare while supporting sustainable farming, respecting consumer rights, and minimizing environmental impact (157).

The shift toward animal-centered farming necessitates support from all stakeholders in the industry, from farmers to retailers and consumers. While many farmers strive to balance animal welfare with economic productivity, the broader transition to ethical farming practices requires alignment across the supply chain. Recent studies (119), have shown growing consumer demand for humanely raised meat, eggs, and dairy products, with many consumers willing to pay a premium for these goods. However, consumer decision-making is often influenced by price, as shown in the research by Yue et al. (158), which found that while interest in sustainable food choices is rising, price remains a critical factor at the point of purchase.

The role of retailers and wholesalers in promoting higher welfare products is crucial. Trewern et al. (159) emphasize the need for systemic changes in food production and distribution, including the willingness of retailers to stock and promote animal welfare-certified products. This may also involve absorbing some of the added costs associated with ethical practices. Liu et al. (127) further underscore the complexity of consumer perceptions, noting that while concern for animal welfare is evident, price and convenience often drive purchasing decisions. This highlights the importance of educating consumers about the benefits of ethically produced food and ensuring transparency in the production chain.

To capitalize on the added value of improved animal welfare, a strategic approach is required. Transparent labeling systems that clearly communicate welfare standards can help inform consumer choices. Marketing strategies that effectively convey the benefits of higher welfare products are necessary to justify premium pricing. Additionally, encouraging retailers to commit to sourcing and promoting welfare-certified products, potentially through policy incentives or consumer advocacy, can help drive change. Innovative pricing models that distribute the costs of welfare improvements more equitably across the supply chain could further support this transition.

HACI technologies present a complex ethical landscape that requires ongoing dialog between ethicists, animal welfare scientists, farmers, and policymakers. As HACI continues to evolve, regular reassessment of its ethical implications will be essential to align its development with our evolving moral responsibilities toward farm animals and the environment.

### Balancing innovation and ethics—critical perspectives on the limitations of HACI in farm animal welfare

While HACI technologies offer promising advancements in farm animal welfare, there are significant limitations and potential risks that must be considered. Over-reliance on technology could inadvertently reduce essential human-animal interaction, which remains a cornerstone of animal care (160). The automation of monitoring and decision-making could desensitize farmers to the more nuanced aspects of animal welfare that are best detected through direct observation. This raises ethical concerns about whether HACI systems, in seeking to maximize efficiency, may shift focus away from the empathetic relationships that form between farmers and animals, which have long been associated with better welfare outcomes (161).

Another potential limitation is the risk of HACI systems misinterpreting behavioral cues, leading to inappropriate interventions. For example, an automated system may detect a reduction in a cow’s movement and interpret it as a sign of stress, triggering environmental changes that may not align with the animal’s actual needs. Such misinterpretations could result in unnecessary interventions that disrupt the animal’s natural behavior and cause more harm than good (162). Furthermore, the high cost of implementing HACI systems could outweigh the benefits, particularly for small-scale farms. While HACI systems can be effective in larger commercial operations, they may impose financial burdens on smaller farms, potentially diverting resources from more traditional welfare-improving strategies, such as increasing staff for animal care or providing more space per animal (163). In these instances, technology could also contribute to increased stress if the automation of care misaligns with an animal’s natural behaviors—such as providing enrichment that fails to engage pigs’ rooting behaviors or offering automated grooming systems that are not used by all cows equally (164).

These critical considerations underscore the need for a balanced approach in adopting HACI. While technology holds the potential to enhance welfare, it should be thoughtfully integrated into farm practices, with attention to both the biological needs of the animals and the socio-economic constraints of the farming system. This nuanced understanding of HACI adoption is vital to avoid negative consequences and to ensure that the benefits of these technologies are maximized without compromising the human-animal bond or the ethical responsibilities of animal care.

### Future directions and challenges

Looking forward, the continuous development and refinement of HACI technologies must consider the complex dynamics of farm environments. The aim should be not only to enhance the physical health of farm animals but also to enrich their psychological well-being (affective states), by fostering environments where animals can thrive. Innovations such as facial recognition for emotion assessment and natural language processing for better understanding of animal communication could revolutionize our interactions with farm animals, making these interactions more intuitive and responsive. By embracing these technologies and the principles of HACI, the future of farming can shift toward more empathic and scientifically informed practices that recognize animals not just as production units but as beings with inherent value and individual interests. This holistic approach promises to reshape the landscapes of animal welfare and farming, creating systems where animal welfare and farm productivity are not at odds but are harmoniously integrated. The integration of HACI can also provide an abundance of new and meaningful data that could aid consumer education in farming practices and make progress in building public trust in the food animal industry.

## Conclusion

HACI technologies offer significant promise for transforming farm animal welfare by leveraging advanced AI, sensor, and interactive enrichment systems to improve health, reduce stress, and enhance cognitive engagement. However, the successful adoption of these technologies requires careful consideration of economic, ethical, and practical factors. Balancing innovation with the need for human-animal interaction and ethical responsibilities is key to ensuring the welfare of farm animals while supporting sustainable agricultural practices. By aligning technological advancements with animal-centered farming and promoting collaboration between stakeholders, HACI technologies have the potential to advance both animal welfare and operational efficiency, meeting the growing consumer demand for ethically produced food. The future of HACI is promising, but its implementation must be guided by a commitment to animal welfare, transparency, and ethical standards, ensuring that it contributes to more humane, productive, and sustainable farming systems.

## Data Availability

The original contributions presented in the study are included in the article/supplementary material, further inquiries can be directed to the corresponding author/s.
